# Supplementary material for: Structural mechanism of anti-MHC-I antibody blocking of inhibitory NK cell receptors in tumor immunity
Source: Commun Biol. 2026 Feb 2;9:350. doi: 10.1038/s42003-026-09641-8 (PMC12966442; doi:10.1038/s42003-026-09641-8)
Supplement: Supplementary file 1 — Supplementary Information [file 42003_2026_9641_MOESM1_ESM.pdf]

Jiang et al. Structural mechanism of anti-MHC-I antibody blocking of inhibitory NK cell receptors in tumor immunity

SUPPLEMENTARY FIGURES AND TABLES

Supplementary Fig. 1 | Binding affinity comparison, B1.23.2 specificity, and Fluorescence staining of transfectants with B1.23.2.

Supplementary Fig. 2 | A protocol for map resolution improvement using cryoSPARC.

Supplementary Fig. 3 | B1.23.2Ab analysis reveals Fc-like Domains.

Supplementary Fig. 4 | cryoEM structure of B1.23.2Fab + HLA-B44:05.

Supplementary Fig. 5 | Peptide variants at position 8 influence affinity of B1.23.2 for HLA-B*44:05.

Supplementary Fig.6 | X-ray structure and comparison with Cryo-EM structures.

Supplementary Fig. 7 | Overlapping contact residues on MHC-I by TCR or KIR with the epitopes of B1.23.2, and a model of competition.

Supplementary Fig. 8 | Molecular Dynamics simulations with mutations of four epitope residues on $\alpha 2_1$ helix of HLA-B*44:05 in binding to B1.23.2.

Supplementary Fig. 9 | Gating strategy for cultured live human PBMC.

Supplementary Fig. 10 | Gating strategy for cultured live human T cells and NK cells.

Supplementary Fig. 11 | Gating strategy for cultured live human monocytes.

Supplementary Fig. 12 | Gating strategy for TIL-derived live lymphocyte singlets.

Supplementary Table 1 | Encoded amino acid sequences of B1.23.2 and HLA-B*44:05.

Supplementary Table 2 | Contact tables.

a

Complex	Binding affinity K_D (μM)	Reference
HLA-B*44:05 to B1.23.2	0.020	This paper
HLA-B*57:01 to KIR3DL1	17.00	Vivian et al.
HLA-C*03:04 to KIR2DL2	9.50	Boyington et al.

b

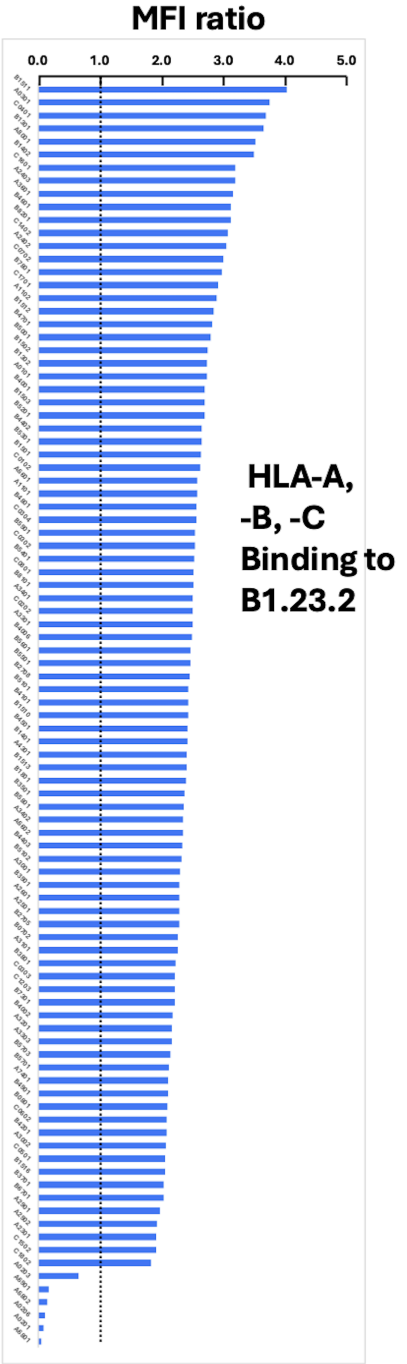

c

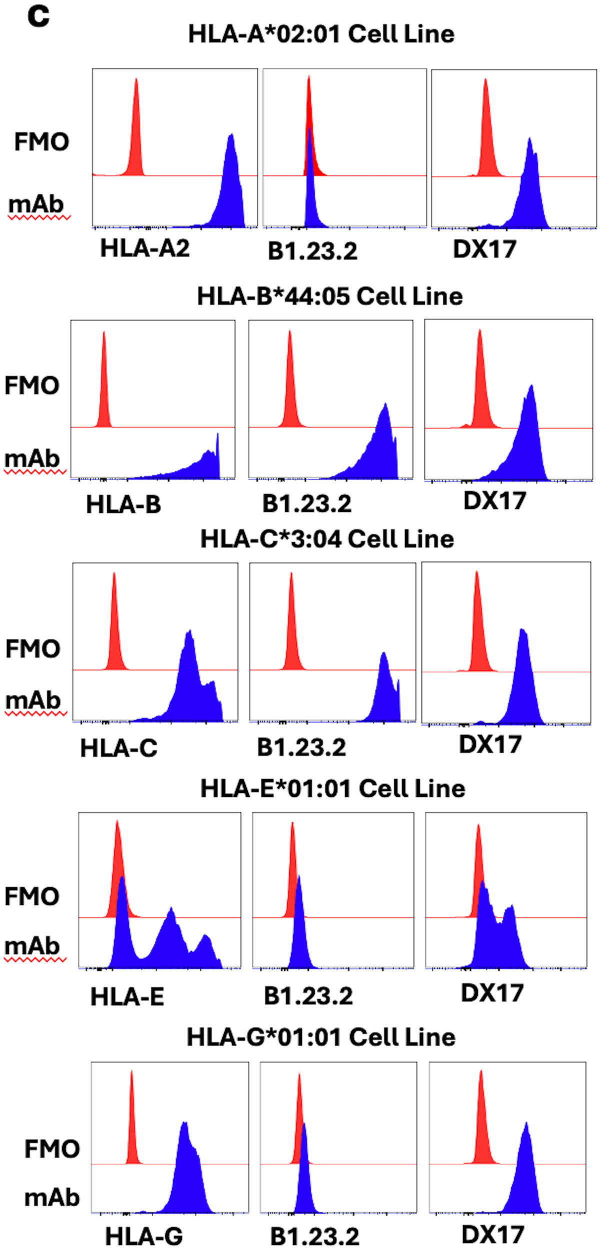

Supplementary Fig. 1

Supplementary Fig. 1 | Binding affinity comparison, B1.23.2 specificity, and fluorescence staining of transfectants with B1.23.2. a. Comparison of binding affinity of B1.23.2 to HLA with KIRs that bind to various HLA **b.** Binding of B1.23.2 to panel of Single Antigen Beads (SABs) screening as described in Methods. B1.23.2 binds all HLA-A, -B, -C relative to W6/32 except, HLA-A*02:01, -A*02:03, -A*02:06, -A*68:01, -A*68:02 and -A*69:01. (Data are plotted as the ratio of the mean of duplicate fluorescence measurements of binding of B1.23.2 to W6/32). Source data are in Supplementary Data 1. **c.** Fluorescence staining of transfectants expressing only the indicated HLA molecules. HeLa cells that were knocked out for endogenous expression of HLA-A, -B, and -C were transfected with cDNAs encoding the indicated HLA glycoproteins and then stained with allele-specific mAbs as described in Methods.

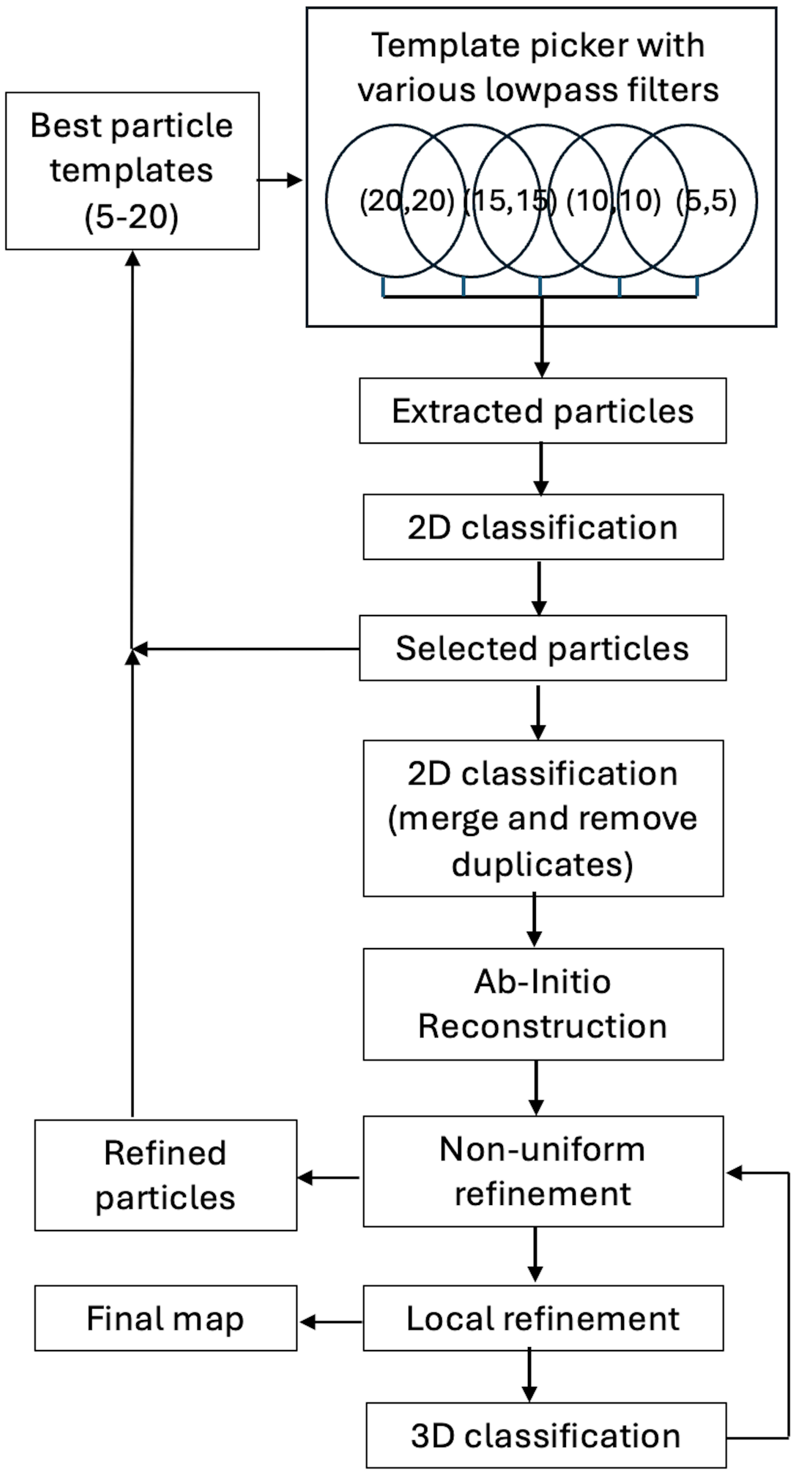

Supplementary Fig. 2

Supplementary Fig. 2 | A protocol for map resolution improvement using cryoSPARC. In the Template picker, with various Lowpass filters (LPF) applied to the template particle and micrograph, multiple runs are submitted in parallel. Then, particles with individual 2D classification are extracted and 2D classification is performed. Selected particles are iteratively cycled. A second round of 2D classification with all previously selected particles is merged and duplicates are removed. In the ab-initio reconstruction, following standard Non-uniform and local refinement, a 3D classification is applied to remove some low-resolution classes.

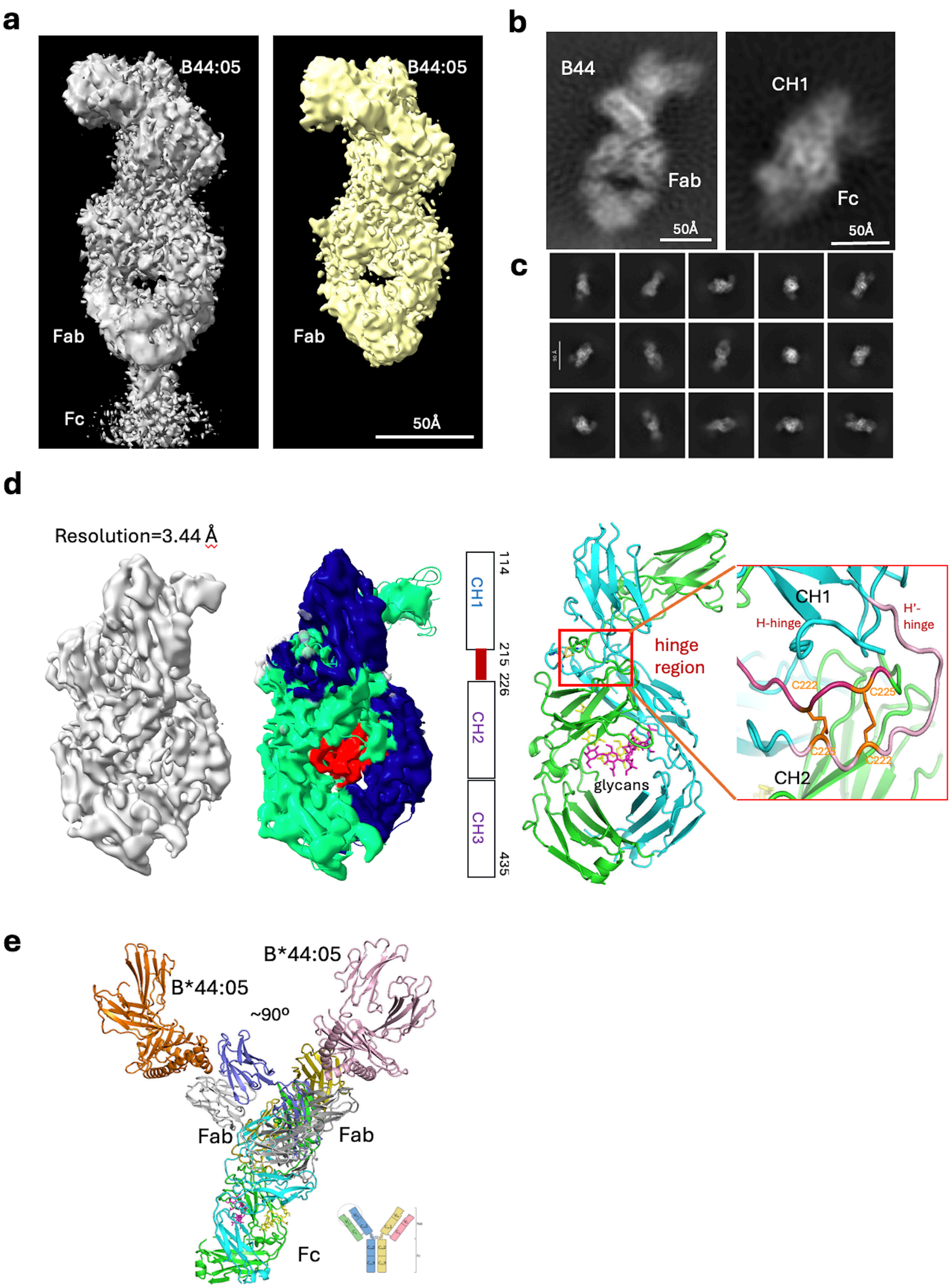

Supplementary Fig. 3

Supplementary Fig. 3 | B1.23.2Ab analysis reveals Fc-like Domains. a. Comparison of the map of B1.23.2 mAb+B*44:05 and B1.23.2 Fab+B*44:05 at the same contour level (0.02 e/Å). **b.** Two types of particles were observed: Fab+B44-like and Fc-like. The Fc domain has an extension that might be part of the C_{H1} domain. **c.** 2D classes for Fc-like particles (selected representatives). **d.** The Fc-like map (3.44Å resolution with 251,084 particles) was fit to the model. Two H-chains are colored blue and cyan, and red indicates the glycans. The model presents the partial C_{H1} domain, the disordered hinge region, and two twisted C_{H1} domains; the hinge loops "cross-over" to form two disulfide bridges. The glycans are linked to N293 of the C_{H2} domain. **e.** A reconstructed model of full-length of B1.23.2 with B*44:05 shows the hinge angle of the two Fabs to be 90º -100º, however, the light chain (gray) clashes at the hinge region.

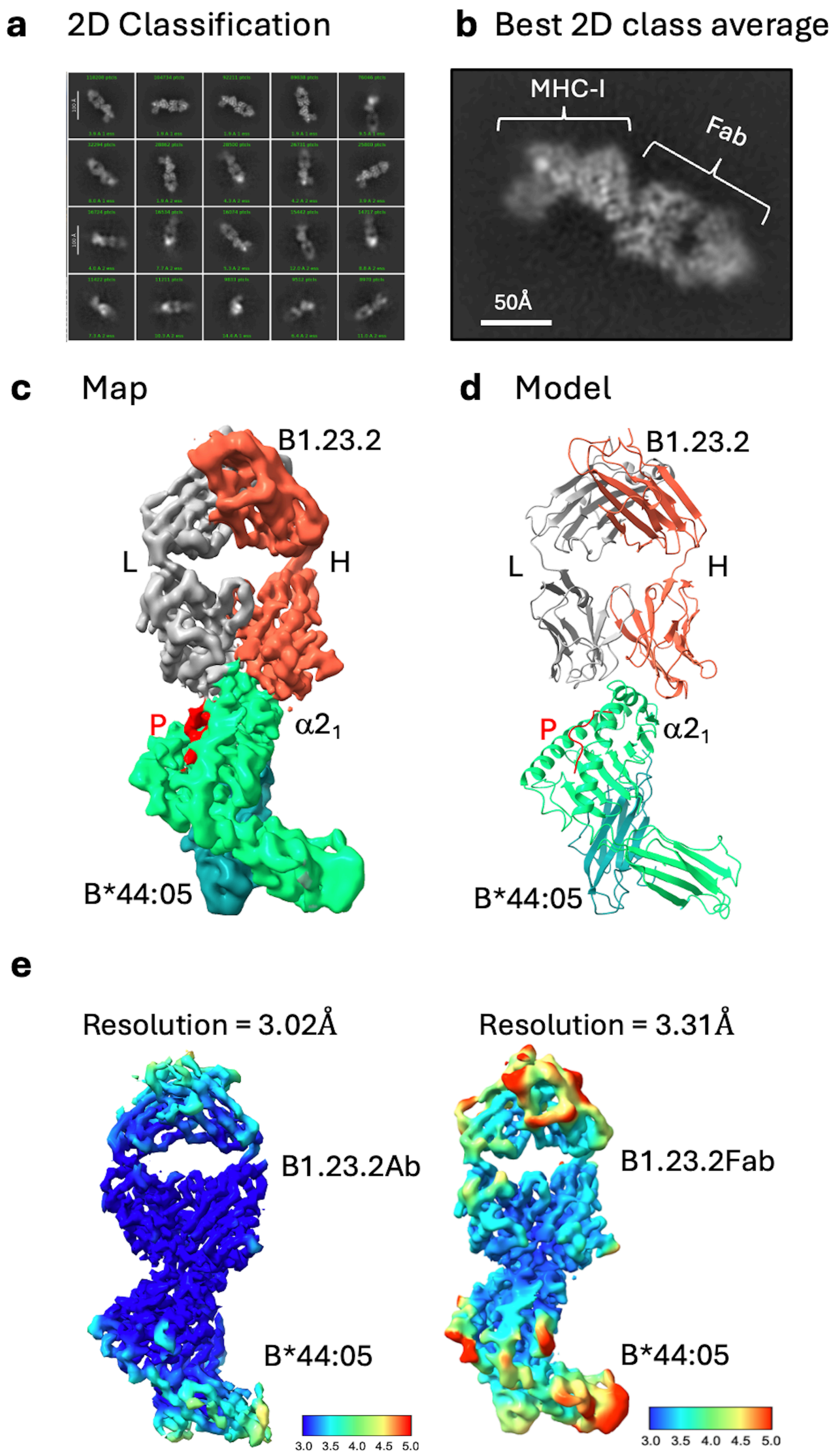

Supplementary Fig. 4

Supplementary Fig. 4 | cryoEM structure of B1.23.2Fab + HLA-B44:05. a. 2D classification after several runs of particle pickings. **b.** The best 2D class clearly shows domains of Fab and MHC-I. **c.** The final refined map at 3.31 Å resolution, colored with their domains, red indicates the peptide. **d.** The refined model (PDB ID: 9D74) is compared with the map, with the same color indicating each domain and peptide. **e.** Local resolution maps reveal the best resolution Ab/HLA-B*44:05 interface. Left: complex of B1.23.2Ab and HLA-B*44:05, map-resolution = 3.02Å. Right: complex of B1.23.2Fab and HLA-B*44:05, map-resolution = 3.31Å. Blue is high resolution; red, low resolution. The color scale bar indicates the resolution distribution.

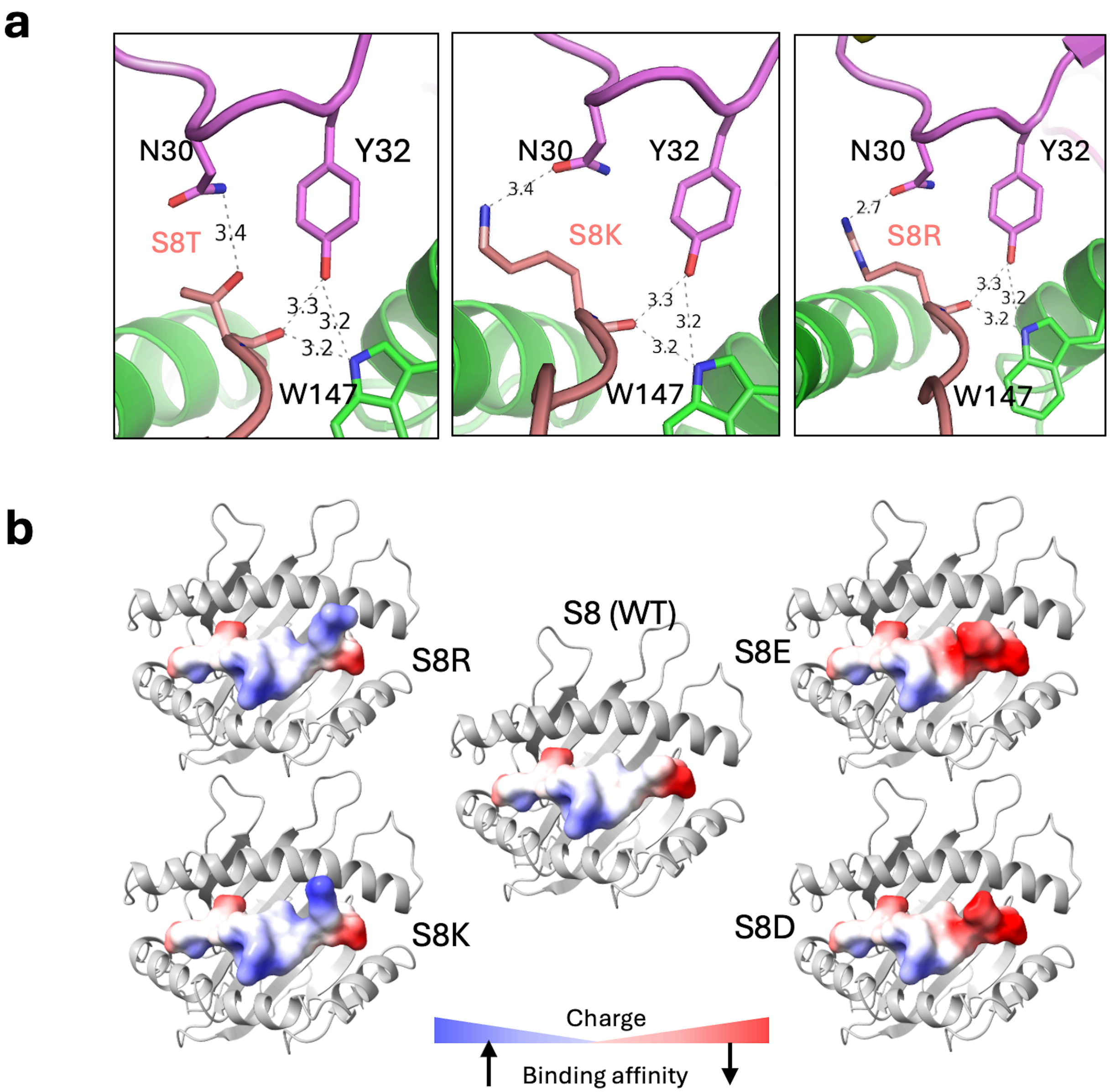

Supplementary Fig. 5

Supplementary Fig. 5 | Peptide variants at position 8 influence affinity of B1.23.2 for HLA-B*44:05. a. Energy-minimized models of peptide variants S8T, S8K and S8R show the formation of the hydrogen bonds with the sidechain of N30 of B1.23.2. **b.** Electrostatic surface charge for S8K, S8R, S8E, and S8D is shown, illustrating that greater positive charge increases the binding affinity, while negative charge decreases the binding affinity, as indicated. Amino acid S8 was changed to each of the indicated amino acids, and energy-minimized in PHENIX. Surface electrostatic charge of the parental and variant peptides was qualitatively displayed using PyMOL. Based on binding data of complexes containing the substituted peptides, measured by SPR as summarized in Fig. 3b, substitution with R or K resulted in higher affinity (lower K_D) and with D or E with lower affinity (higher K_D). The schematic indicates the qualitative relationship between surface charge in the vicinity of position 8 (blue=basic, red=acidic) and the corresponding change in binding affinity.

a **b**

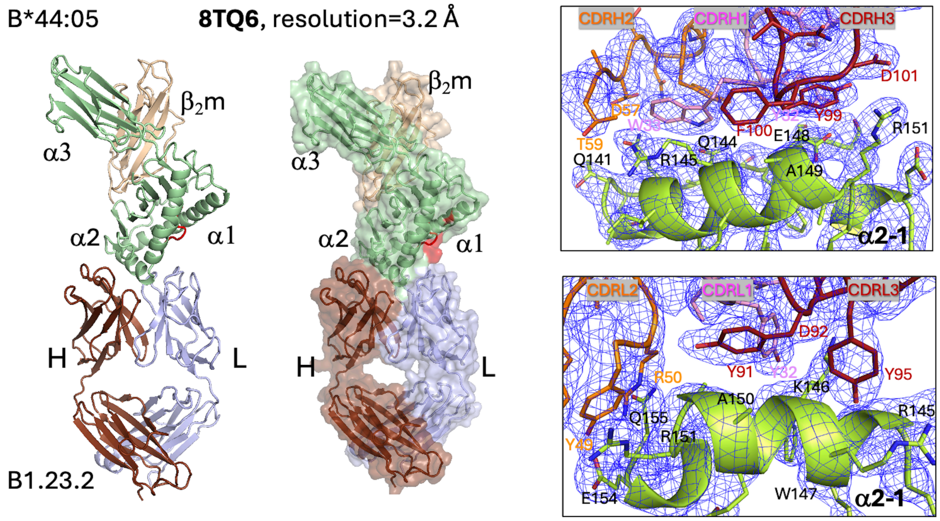

c

mAb+MHC-I	Cryo-EM Resolution	PDB ID	BSA (Å²)	Mean B-value (Å²)		X-ray Resolution	PDB ID	BSA (Å²)	Mean B-value (Å²)	RMSD (MHCI/Fab) (Å)
B1.23.2 Fab+B*44:05	3.3 Å	**9D74**	931	172.4		3.2 Å	**8TQ6**	890	47.7	1.6 (1.5/1.3)
B1.23.2 mAb+B*44:05	3.0 Å	**9D73**	899	128.8		3.2 Å	**8TQ6**	890	47.7	1.5 (1.4/1.3)

Supplementary Fig.6

Supplementary Fig.6 | X-ray structure and comparison with Cryo-EM structures. a. X-ray crystal structure of Fab of B1.23.2 in complex with HLA-B*44:05 (PDB-ID: 8TQ6). **b**. Electron density maps of X-ray crystal structure of Fab of B1.23.2 in complex with HLA-B*44:05 (PDB ID: 8TQ6), [2mFo-DFc map contoured at 2.5 sigma]: top panel shows that CDR loops of the H chain interact with the $\alpha2_1$ helix, and lower panel shows CDR loops of the L chain interacting with the $\alpha2_1$ helix. **c**. The differences in RMSD and BSA between the X-ray crystal and the two cryo-EM structures.

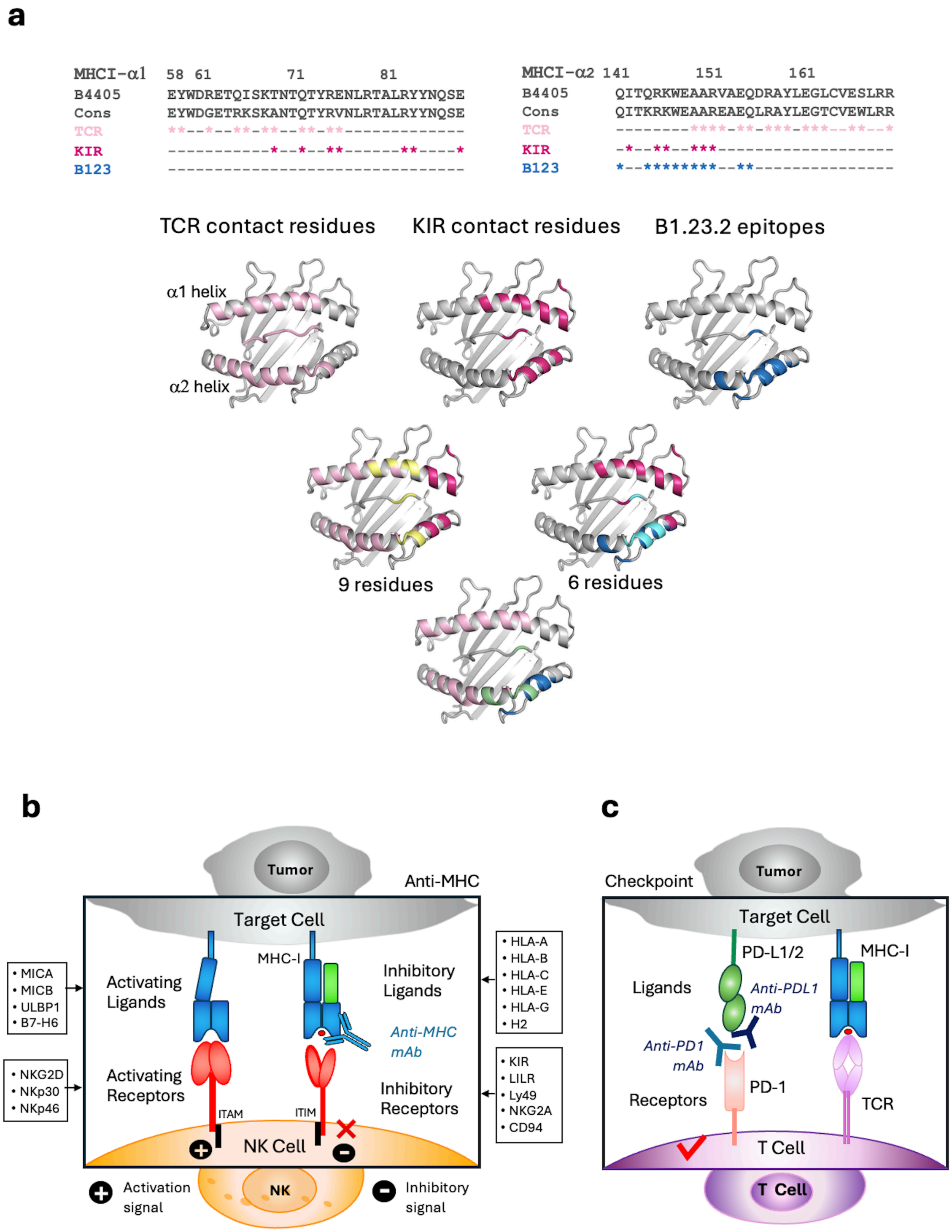

Supplementary Fig. 7

Supplementary Fig. 7 | Overlapping contact residues on MHC-I by TCR or KIR with the epitopes of B1.23.2, and a model of competition. a. In the top panel, alignment of the epitopes (contact residues or binding sites to HLA-B*44:05 and the consensus sequences. Stars indicate contact; dash does not, pink indicates TCR (warn pink indicates KIR, and blue indicates B1.23.2. Yellow means the overlap between TCR and KIR, cyan means the overlap between KIR and B1.23.2, and pale green represents overlap between TCR and B1.23.2. KIR contacts two peptide residues at P7 and P8, B1.23.2 contacts only one residue at P8, while TCR contacts all eight peptide residues from P1 to P8. TCR contact residues are from the majority of abTCR (representative PDB IDs: 10GA, 1AO7, 1BD2, 1LP9, 2BNQ, 3HG1, 3GSN, Roomp *et al., **Mol. Immuno.*** 2011). KIR contact residues are from KIR2DL2 (PDB ID: 1EFX) and KIR3DL1 (PDB ID: 3VH8). **b.** A generalized mechanistic model of inhibitory receptor and anti-MHC-I mAb competition for binding to MHC-I. When the anti-MHC mAb binds to MHC-I, it blocks the interaction between MHC-I and the inhibitory receptors (KIRs/LILRs/Ly49s), which may lead to cancelling the inhibitory signals, and enhancing the activation signals, resulting in the downstream killing and tumor suppression. **c**. An illustration of anti-PD1/PD-L1 mAb in the checkpoint pathway. When the anti-PD-1/PD-L1 mAb blocks the interaction between PD-1 and PD-L1, it leads to the activation of T cell activity, leading to tumor cell death (Parvez *et al.* ***Frontiers in Immunology***, 2023).

a

```
                131        141        151
                |          |          |
HLA-B*44:05   SSWTAADTAAQITQRKWEAARVAEQ
HLA-A*02:01   RSWTAADMAAQTTKHKWEAAHVAEQ
```

I142T
Q144K
R145H
R151H

b

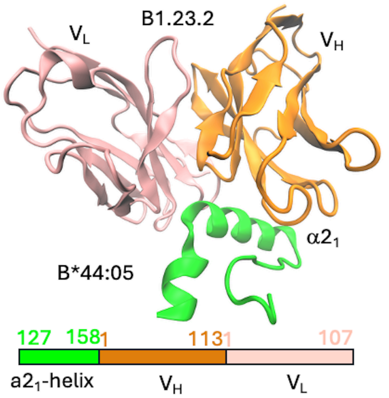

c Probability distribution of RMSD for B*44:05 and mutants

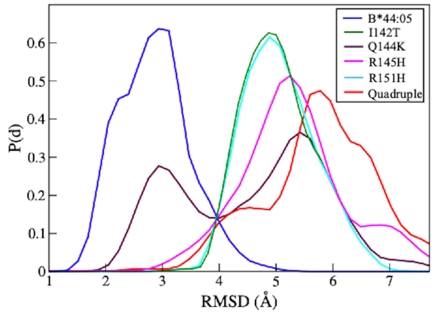

d Average RMSD on B*44:05 and mutants

Mutants	mean RMSD (μ, Å)	Std Dev (σ, Å)
B*44:05(α2$_1$)	2.96	0.64
I142T	4.88	0.66
R151H	4.88	0.66
R145H	5.23	0.66
Q144K	2.93, 5.41	1.54
I142T/Q144K/R145H /R151H (Quadruple)	5.78	0.97

e RMSF of α2$_1$-helix

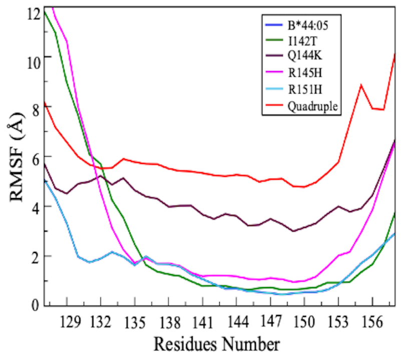

f RMSF of variable heavy chain (V$_H$)

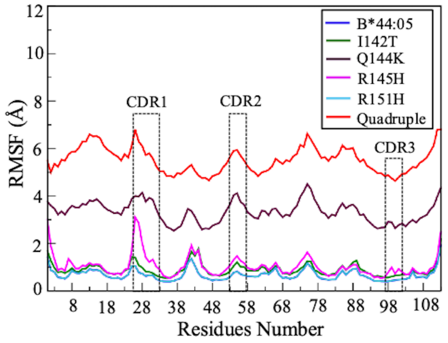

g RMSF of variable light chain (V$_L$)

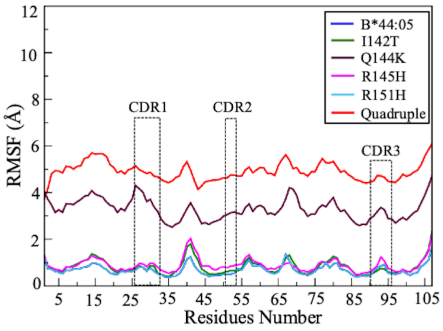

Supplementary Fig. 8

Supplementary Fig. 8 | Molecular Dynamics simulations with mutations of four epitope residues on $\alpha 2_1$ helix of HLA-B*44:05 in binding to B1.23.2. a. Sequence of the $\alpha 2_1$ helix (residues 131-155) of HLA-B*44:05 aligned with HLA-A*02:01. Colors (cyan to yellow) indicate positions where substitutions were introduced. **b.** Ribbon view of $\alpha 2_1$ fragment of HLA-B*44:05 (residues 127-158), V_H (residues 1-113, orange), and V_L (residues 1-107, pink) of B1.23.2 mAb. **c.** Conformational probability distribution (P(d)) of RMSD values for the HLA-B*44:05 and its mutants. The distribution shows the differences in conformational flexibility among the mutants compared to the HLA-B*44:05. HLA-B*44:05 shows a narrow and sharply peaked distribution, indicating stable structural conformation throughout the simulation. In contrast, the other mutants, especially Q144K and quadruple, exhibit broader distributions or shifted peaks, reflecting increased conformational flexibility and possible structural deviation from native structure. **d.** The mean RMSD and SD values for B*44:05 and mutations. The large average RMSD indicates the dissociation of the mutants, particularly of the Quadruple that agreed with the mutagenesis experiments in Fig.3d. **e.** RMSF analysis of the $\alpha 2_1$ fragment of HLA-B*44:05 and its mutants. **f.** RMSF of the variable heavy (V_H) chain of B1.23.2 in complex with HLA-B44:05 and its mutants. **g.** RMSF of the variable light (V_L) chain of B1.23.2 in complex with HLA-B44:05 and its mutants. RMSF values represent the positional flexibility of each residue over the course of molecular dynamics simulations.

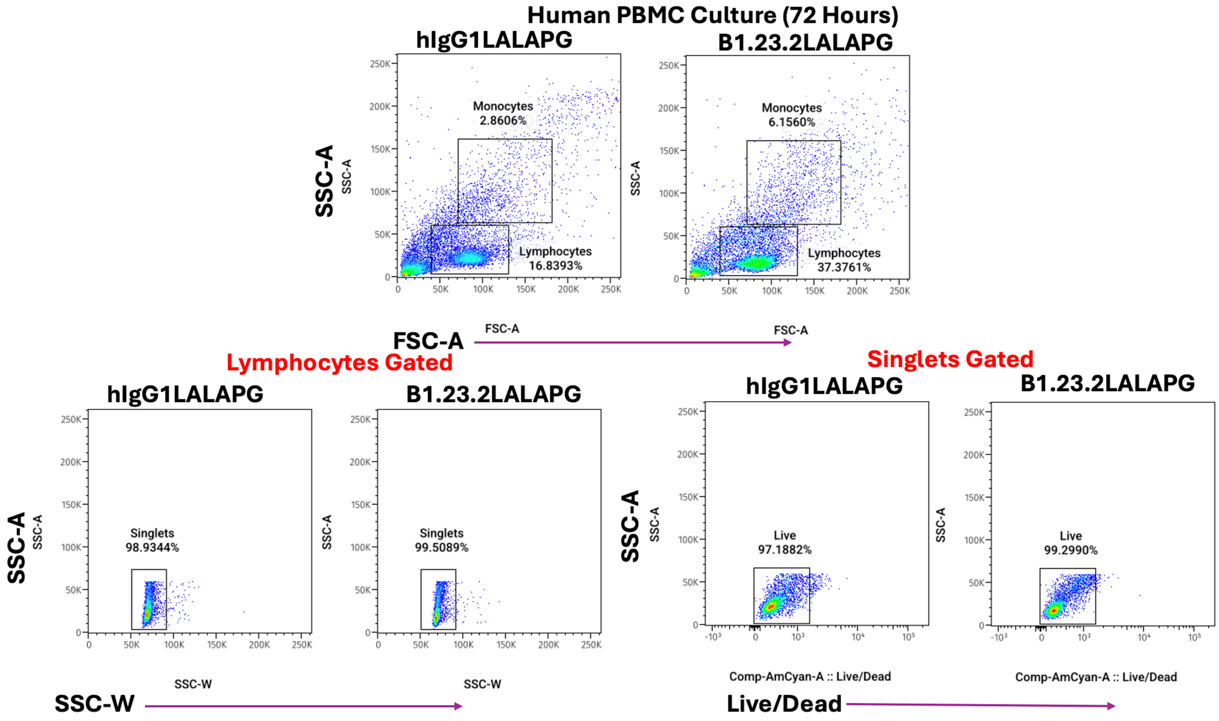

Supplementary Figure 9.

Supplementary Fig. 9 | Gating strategy for cultured live human PBMC. Lymphocyte and singlets, based on scattering as indicated.

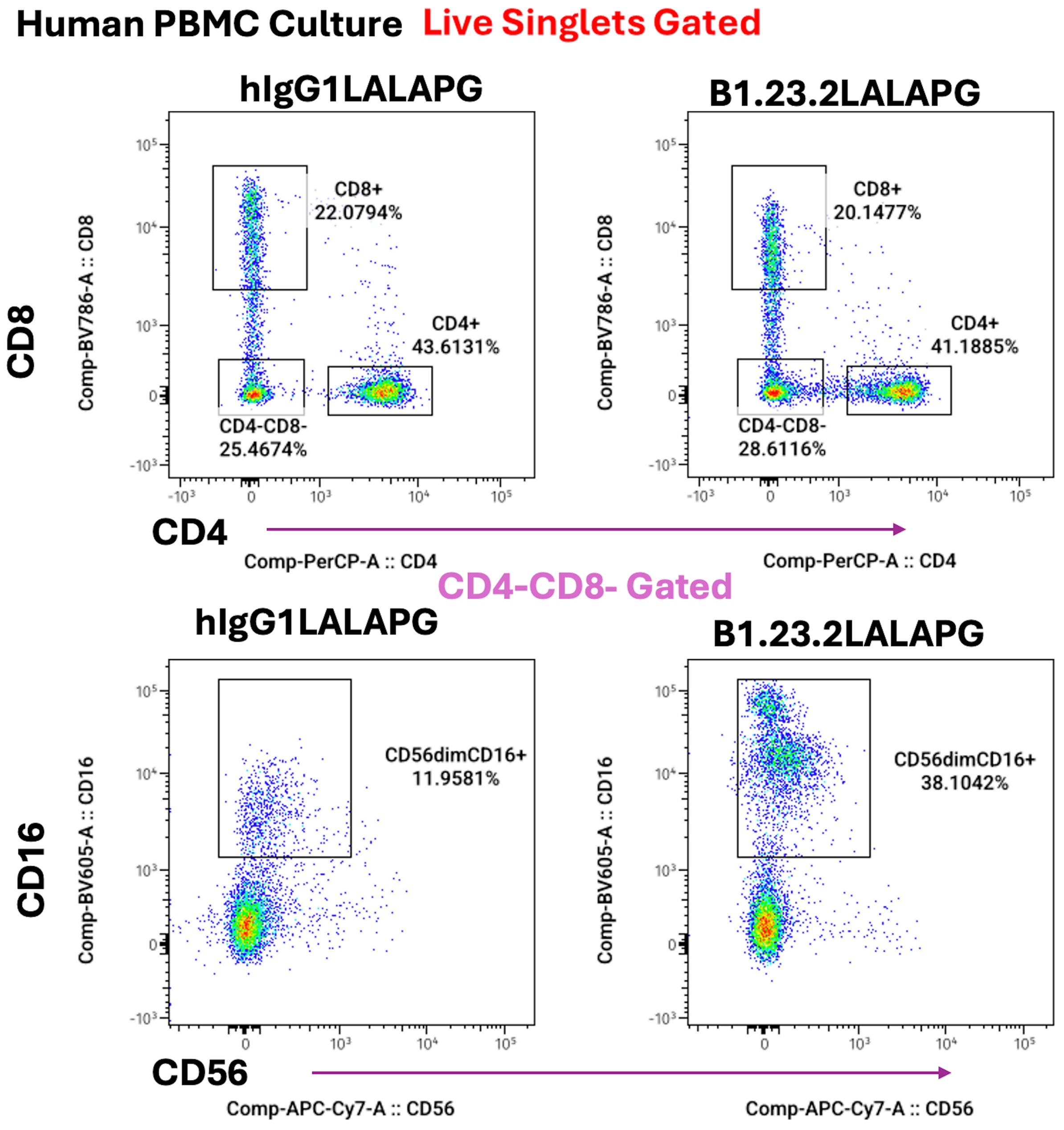

Supplementary Figure 10.

Supplementary Fig. 10 | Gating strategy for cultured live human T cells and NK cells. Gating based on CD4, CD8 CD16 and CD56 and stained as indicated.

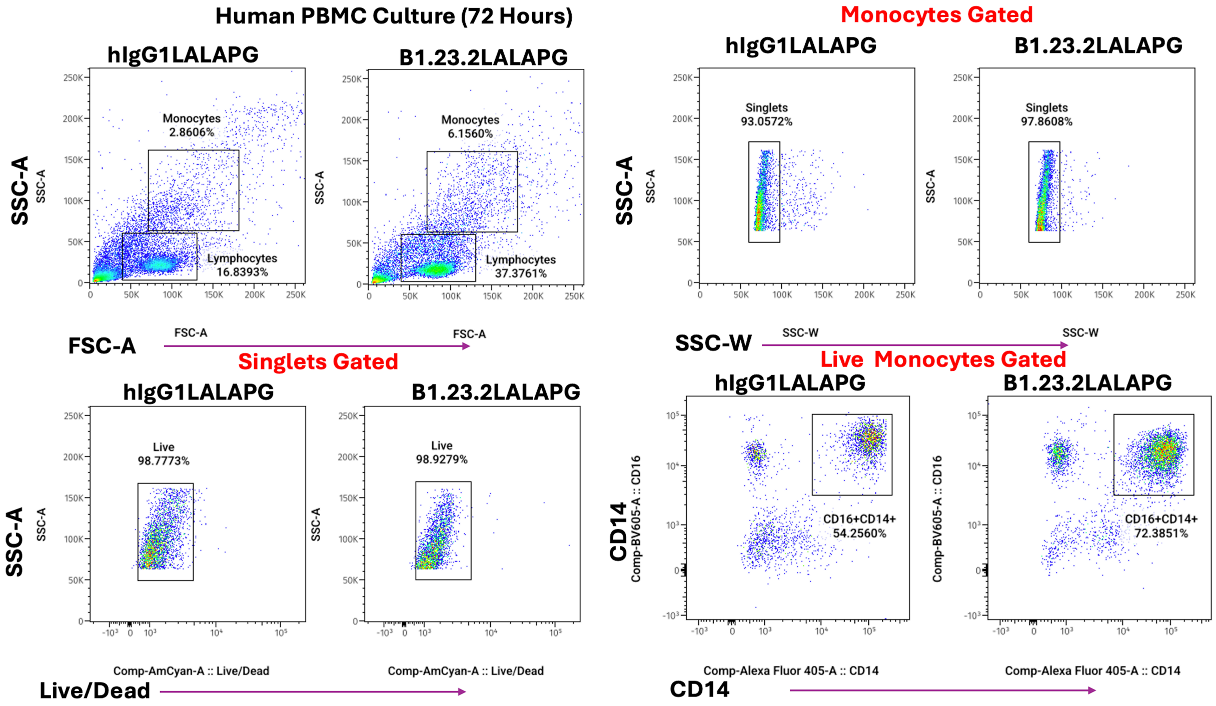

Supplementary Figure 11.

Supplementary Fig. 11 | Gating strategy for cultured live human monocytes. Gating based on scattering and CD14 expression as indicated.

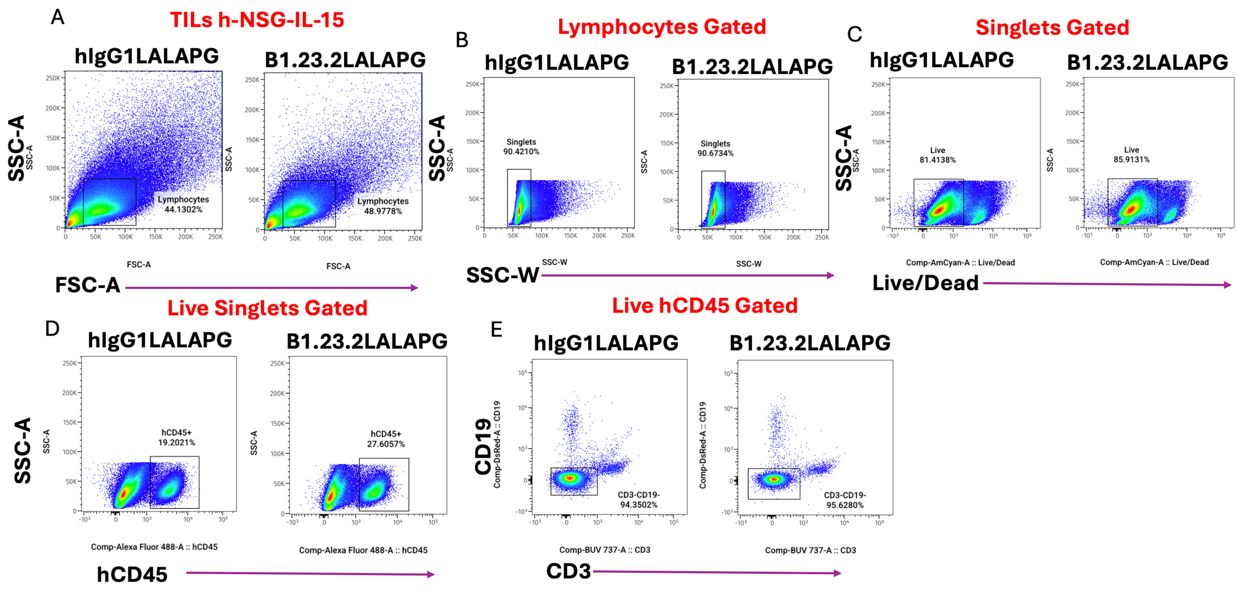

Supplementary Figure 12.

Supplementary Fig. 12 | Gating strategy for TIL-derived live lymphocyte singlets. Gating based on scattering and indicated markers.

Sequences of B1.23.2 and HLA-B*44:05 (CDR1, CDR2, CDR3)*
**>B1.23.2 Fab VH in black, CH1 domain of mouse IgG2a in blue**<br>QVQLQQSGTVLARPGSSVKMSCKASGYSFTSYWMHWVKQRPGQGLEWIGAIYPGNSDATYNQKFKGKAKLTAVTSANTAY<br>MELSSLTNEDSAVYYCTNYFDQWGQGTTLTVSSAKTTAPSVYPLAPVCGDTTGSSVTLGCLVKGYFPEPVTLTWNSGSLS<br>SGVHTFPAVLQSDLYTLSSSVTVTSSTWPSQSITCNVAHPASSTKVDKKIE
**>B1.23.2 Fab VL in black, C kappa in blue**<br>DIQMTQTPSSMYASLGERVTITCKASQDINSYLNWFQLKPGKSPKTLIYRANRLVDGVPSRFSGSGSGQDYSLTISSLEY<br>EDMGIYYCLQYDELYTFGGGTKLEMKRADAAPTVSIFPPSSEQLTSGGASVVCFLNNFYPKDINVKWKIDGSERQNGVLN<br>SWTDQDSKDSTYSMSSTLTLTKDEYERHNSYTCEATHKTSTSPIVKSFNRNEC
**>B1.23.2 mAb VH in black, CH1 domain of human IgG1 in blue, hinge in green, plus Fc in purple**<br>QVQLQQSGTVLARPGSSVKMSCKASGYSFTSYWMHWVKQRPGQGLEWIGAIYPGNSDATYNQKFKGKAKLTAVTSANTAY<br>MELSSLTNEDSAVYYCTNYFDQWGQGTTLTVSSASTKGPSVFPLAPSSKSTSGGTAALGCLVKDYFPEPVTVSWNSGALT<br>SGVHTFPAVLQSSGLYSLSSVVTVPSSSLGTQTYICNVNHKPSNTKVDKKVEPKSCDKTHTCPPCPAPEAAGGPSVFLFP<br>PKPKDTLMISRTPEVTCVVVDVSHEDPEVKFNWYVDGVEVHNAKTKPREEQYNSTYRVVSVLTVLHQDWLNGKEYKCKVS<br>NKALGAPIEKTISKAKGQPREPQVYTLPPSREEMTKNQVSLTCLVKGFYPSDIAVEWESNGQPENNYKTTPPVLDSDGSF<br>FLYSKLTVDKSRWQQGNVFSCSVMHEALHNHYTQKSLSLSPGK
**>HLA-B*44:05 A-chain (Epitopes contacted by B1.23.2)**<br>GSHSMRYFYTAMSRPGRGEPRFITVGYVDDTLFVRFDSDATSPRKEPRAPWIEQEGPEYWDRETQISKTNTQTYRENLRT<br>ALRYYNQSEAGSHIIQRMYGCDVGPDGRLLRGYDQYAYDGKDYIALNEDLSSWTAADTAAQITQRKWEAARVAEQDRAYL<br>EGLCVESLRRYLENGKETLQRADPPKTHVTHHPISDHEVTLRCWALGFYPAEITLTWQRDGEDQTQDTELVETRPAGDRT<br>FQKWAAVVVPSGEEQRYTCHVQHEGLPKPLTLRW
**>HLA-B*44:05 B-chain**<br>MIQRTPKIQVYSRHPAENGKSNFLNCYVSGFHPSDIEVDLLKNGERIEKVEHSDLSFSKDWSFYLLYYTEFTPTEKDEYA<br>CRVNHVTLSQPKIVKWDRDM
**>HLA-B*44:05 Peptide, HLA-DPA1*02:01 derived peptide 77-85**<br>EEFGRAFSF

* CDR loops are defined by IMGT

Supplementary Table 1

Supplementary Table 1 | Encoded amino acid sequences of B1.23.2 and HLA-B*44:05. Sequences of Fab V_H, V_L of the mouse IgG2a as well as the engineered chimeric mouse/human IgG1 V_HC_{H1} molecules are presented in single-letter amino acid code. CDR1, CDR2, and CDR3 of both the V_H and V_L as determined by the IMGT numbering system *(LeFranc,* Nuc. Acids Res *1999)*, are color coded. The sequences of the HLA-B*44:05 heavy (A) and light (B) chains, as well as the antigenic peptide derived from HLA-DPA1*02:01$_{77\text{-}85}$ are shown. Epitope residues that contact with B1.23.2 are shown in firebrick color.

B*44:05/Fab B1.23.2				Distance (Å)		
**B4405**	**B123**	**C**	**CDR**	**9D73**	**9D74**	**8TQ6**
S131	Y32	H	1	3.33	2.97	2.27
S132	Y32	H	1		3.60	3.84
Q141	W33	H	1	3.29	3.52	3.59
Q141	D57	H	2	3.45	3.89	3.42
Q144	W33	H	1	3.01	3.34	3.07
R145	W33	H	1	3.37	3.11	3.32
R145	T59	H	2	3.83	3.73	3.24
E148	Y32	H	1	3.56	3.16	3.17
E148	W33	H	1	2.61	2.71	
E148	Y99	H	3	3.19	3.17	3.19
A149	Y99	H	3	3.62		3.45
R151	F100	H	3	3.28	2.58	3.22
R151	D101	H	3	3.30	3.20	3.04
R145	Y95	L	3	3.13	2.87	3.21
K146	Y32	L	1	3.57	3.68	3.46
K146	Y91	L	3	3.48	3.71	3.13
K146	D92	L	3	2.89	2.65	2.99
W147	Y32	L	1	3.18	3.50	3.55
A149	Y91	L	3	3.72	3.58	3.29
A150	Y32	L	1	3.77	3.76	
A150	Y49	L	2	3.44	3.48	3.31
A150	R50	L	2	3.16	3.88	3.41
A150	Y91	L	3	3.78	3.90	3.49
R151	Y49	L	2	3.71		3.32
R151	R50	L	2	3.32		3.03
E154	Y49	L	2	3.68		2.27
Q155	R50	L	2	3.18		3.05
S8	Y32	L	2	3.09	3.59	3.02

C*03:01+KIR2DL2		Dist (Å)
**Cw3-A**	**KIR2DL2**	**1EFX**
R69	E21	3.16
R69	M70	3.01
Q72	M70	3.50
Q72	D72	3.36
R75	D72	3.90
R75	F45	2.94
V76	F45	3.53
R79	F45	3.17
R79	K44	3.44
N80	K44	3.27
N80	S184	3.69
Y84	D183	3.56
R145	S133	2.76
R145	D135	2.62
K146	Y105	3.33
K146	F181	3.60
K146	D183	2.46
K146	S184	3.74
A149	Y105	3.62
A149	E106	2.93
A149	S132	3.32
A150	L104	3.55
R151	E106	2.73

B*57:01+KIR3DL1		Dist (Å)
**B57-A**	**KIR3DL1**	**3VH8**
G16	S11	3.60
G16	F9	3.49
G16	H29	3.24
G16	F34	3.84
R17	F9	3.45
G18	F9	3.49
E19	F9	3.82
Q72	M165	3.11
E76	A167	3.62
R79	G138	3.28
R79	S140	3.47
I80	L166	3.63
R83	H278	2.74
Y84	H278	3.68
E89	W13	2.99
I142	H278	3.75
R145	S228	2.88
R145	D230	2.56
R145	F276	3.37
K146	Y200	3.45
K146	F276	3.61
K146	S279	2.83
K146	E282	2.69
A149	Y200	3.74
A149	E201	3.32
A149	S227	3.65
A149	F276	3.89
A150	P199	3.85
A150	Y200	3.74
R151	E201	3.68

Supplementary Table 2.

Supplementary Table 2 | Contact tables. List of contacts of HLA-B*44:05+Fab B1.23.2 (9D73, 9D74, 8TQ6); HLA-C*03:01+KIR2DL2 (1EFX); and HLA-B*57:01+KIR3DL1 (3VH8), (distances within 4.0 Å). Contact distances were calculated with PDBsum. Only the closest contacts per residue are shown. Fab chains are indicated by color (H, orange; L, magenta), and complementarity- determining regions (CDR-loops) are noted.
