# Supplementary material for: Structural mechanism of anti-MHC-I antibody blocking of inhibitory NK cell receptors in tumor immunity
Source: Commun Biol. 2026 Feb 2;9:350. doi: 10.1038/s42003-026-09641-8 (PMC12966442; doi:10.1038/s42003-026-09641-8)
Supplement: Supplementary file 2 — Description of Additional Supplementary Materials [file 42003_2026_9641_MOESM2_ESM.docx]

**Description of Additional Supplementary Files**

**File name:** Supplementary Data 1

**Description:** Source Data for Supplementary Figure 1b

**File name:** Supplementary Data 2

**Description:** Source Data for Figure 1a and 3d
