# Supplementary material for: Structural mechanism of anti-MHC-I antibody blocking of inhibitory NK cell receptors in tumor immunity
Source: Commun Biol. 2026 Feb 2;9:350. doi: 10.1038/s42003-026-09641-8 (PMC12966442; doi:10.1038/s42003-026-09641-8)
Supplement: Supplementary file 5 — Reporting Summary [file 42003_2026_9641_MOESM5_ESM.pdf]

nature portfolio

Corresponding author(s): *Double-anonymous peer review submissions: write DAPR and your manuscript number here instead of author names.*

Last updated by author(s): *YYYY-MM-DD*

Reporting Summary

Nature Portfolio wishes to improve the reproducibility of the work that we publish. This form provides structure for consistency and transparency in reporting. For further information on Nature Portfolio policies, see our Editorial Policies and the Editorial Policy Checklist.

Statistics

For all statistical analyses, confirm that the following items are present in the figure legend, table legend, main text, or Methods section.

n/a	Confirmed	
☐	☒	The exact sample size (*n*) for each experimental group/condition, given as a discrete number and unit of measurement
☐	☒	A statement on whether measurements were taken from distinct samples or whether the same sample was measured repeatedly
☐	☒	The statistical test(s) used AND whether they are one- or two-sided<br>*Only common tests should be described solely by name; describe more complex techniques in the Methods section.*
☐	☒	A description of all covariates tested
☐	☒	A description of any assumptions or corrections, such as tests of normality and adjustment for multiple comparisons
☐	☒	A full description of the statistical parameters including central tendency (e.g. means) or other basic estimates (e.g. regression coefficient) AND variation (e.g. standard deviation) or associated estimates of uncertainty (e.g. confidence intervals)
☒	☐	For null hypothesis testing, the test statistic (e.g. *F*, *t*, *r*) with confidence intervals, effect sizes, degrees of freedom and *P* value noted<br>*Give P values as exact values whenever suitable.*
☒	☐	For Bayesian analysis, information on the choice of priors and Markov chain Monte Carlo settings
☒	☐	For hierarchical and complex designs, identification of the appropriate level for tests and full reporting of outcomes
☒	☐	Estimates of effect sizes (e.g. Cohen's *d*, Pearson's *r*), indicating how they were calculated

Our web collection on statistics for biologists contains articles on many of the points above.

Software and code

Policy information about availability of computer code

Data collection	All data collection programs are indicated and referenced: SerialEM for cryo EM; XDS for X-ray diffraction.
Data analysis	All data analysis programs are indicated and referenced: CryoSPARC for cryoEM; Phaser, PHENIX, Coot, and ChimeraX for X-ray.

For manuscripts utilizing custom algorithms or software that are central to the research but not yet described in published literature, software must be made available to editors and reviewers. We strongly encourage code deposition in a community repository (e.g. GitHub). See the Nature Portfolio guidelines for submitting code & software for further information.

Data

Policy information about availability of data

All manuscripts must include a data availability statement. This statement should provide the following information, where applicable:

- Accession codes, unique identifiers, or web links for publicly available datasets
- A description of any restrictions on data availability
- For clinical datasets or third party data, please ensure that the statement adheres to our policy

The cryo-EM maps were deposited in the Electron Microscopy Data Bank under the accession IDs EMD-46601 (3.02 Å), EMD-46602 (3.31 Å) and EMD-70276 (3.44 Å), and the atomic coordinates were deposited in the PDB under the accession ID 9D73, 9D74, and 9OA9. X-ray crystal structure data and atomic coordinates were deposited in PDB under the accession ID 8TQ6.

Research involving human participants, their data, or biological material

Policy information about studies with human participants or human data. See also policy information about sex, gender (identity/presentation), and sexual orientation and race, ethnicity and racism.

Reporting on sex and gender	NA
Reporting on race, ethnicity, or other socially relevant groupings	NA
Population characteristics	NA
Recruitment	NA
Ethics oversight	NA

Note that full information on the approval of the study protocol must also be provided in the manuscript.

Field-specific reporting

Please select the one below that is the best fit for your research. If you are not sure, read the appropriate sections before making your selection.

☒ Life sciences ☐ Behavioural & social sciences ☐ Ecological, evolutionary & environmental sciences

For a reference copy of the document with all sections, see nature.com/documents/nr-reporting-summary-flat.pdf

Life sciences study design

All studies must disclose on these points even when the disclosure is negative.

Sample size	NA
Data exclusions	NA
Replication	NA
Randomization	NA
Blinding	NA

Reporting for specific materials, systems and methods

We require information from authors about some types of materials, experimental systems and methods used in many studies. Here, indicate whether each material, system or method listed is relevant to your study. If you are not sure if a list item applies to your research, read the appropriate section before selecting a response.

Materials & experimental systems

n/a	Involved in the study	
☐	☒	Antibodies
☐	☒	Eukaryotic cell lines
☒	☐	Palaeontology and archaeology
☐	☒	Animals and other organisms
☒	☐	Clinical data
☒	☐	Dual use research of concern
☒	☐	Plants

Methods

n/a	Involved in the study	
☒	☐	ChIP-seq
☐	☒	Flow cytometry
☒	☐	MRI-based neuroimaging

Antibodies

Antibodies used	anti-HLA-C, clone DT-9, BD Biosciences 566372<br>anti-HLA-B,C, clone B2.23.2 (Thermofished 17-5935-42<br>anti-HLA-A,B,C, clone DX17, BD Biosciences # 560169

	B1.23.2: purified from hybridoma obtained from Dr. Bernard LaFont; also expressed and purified from Expi293S cells by transfecting H and L chain encoding plasmids.<br>anti-HLA-A2, clone BB7.2, BD Biosciences # 568757<br>anti-HLA-B, clone YTH 76.3, BD Biosciences #567211<br>anti-HLA-C, clone DT-9, BD Biosciences 566372<br>anti-HLA-B,C, clone B2.23.2 (Thermofished 17-5935-42<br>anti-HLA-A,B,C, clone DX17, BD Biosciences # 560169
Validation	all antibodies behaved as previously reported with respect to allelic specificity.

Eukaryotic cell lines

Policy information about cell lines and Sex and Gender in Research

Cell line source(s)	HeLa, ATCC # CCL-2<br>KLM1,<br>(RRID:CVCL_5146)
Authentication	HLA typing of cells was consistent with previous designations.
Mycoplasma contamination	HeLa was not tested-used for protein production. KLM1 was tested and negative for mycoplasma
Commonly misidentified lines<br>(See ICLAC register)	*Name any commonly misidentified cell lines used in the study and provide a rationale for their use.*

Animals and other research organisms

Policy information about studies involving animals; ARRIVE guidelines recommended for reporting animal research, and Sex and Gender in Research

Laboratory animals	Mus musculus-C57BL/6
Wild animals	*Provide details on animals observed in or captured in the field; report species and age where possible. Describe how animals were caught and transported and what happened to captive animals after the study (if killed, explain why and describe method; if released, say where and when) OR state that the study did not involve wild animals.*
Reporting on sex	Sex was not considered in study design
Field-collected samples	N/A
Ethics oversight	All animal protocols used in<br>this study were approved by the National Institute of Allergy and Infectious Diseases Animal Care and Use Committee (Protocol # LISB 15E).

Note that full information on the approval of the study protocol must also be provided in the manuscript.

Plants

Seed stocks	N/A
Novel plant genotypes	N/A
Authentication	N/A

Flow Cytometry

Plots

Confirm that:

☒ The axis labels state the marker and fluorochrome used (e.g. CD4-FITC).

☒ The axis scales are clearly visible. Include numbers along axes only for bottom left plot of group (a 'group' is an analysis of identical markers).

☒ All plots are contour plots with outliers or pseudocolor plots.

☒ A numerical value for number of cells or percentage (with statistics) is provided.

Methodology

Sample preparation	For surface staining, HeLa cells (1 x 106) that express individually tranfected HLA molecules, in staining buffer (PBS, 10% heat-inactivated FBS, and 0.05% sodium azide), were incubated with the following surface conjugate antibodies in 1:50 dilutions: anti-HLA-A2 (mAb BB7.2, BD Biosciences 568757) , Anti-HLA-B (mAb YTH 76.3, BD Biosciences 567211) , Anti-HLA-C (DT-9, BD Biosciences 566372) , Anti-HLA-E (3D12, BD Biosciences 567418), Anti-HLA-G (87G, Biolegend 335912), B1.23.2 (ThermoFisher 17-5935-42) and DX17 (BD Biosciences 560169) in the presence of Fc block (BD biosciences).
Instrument	LSRFortessa (BD Biosciences) flow cytometer
Software	FASCDiva software and analyzed by FlowJo (tree star version 10) software.
Cell population abundance	No sorting.
Gating strategy	Gating will be supplied as additional supplemental figure.

☐ Tick this box to confirm that a figure exemplifying the gating strategy is provided in the Supplementary Information.
